# Melanin‐based ornament darkness positively correlates with across‐season nutritional condition

**DOI:** 10.1002/ece3.6898

**Published:** 2020-10-12

**Authors:** Gergely Hegyi, Miklós Laczi, Dóra Kötél, Tibor Csörgő

**Affiliations:** ^1^ Department of Systematic Zoology and Ecology ELTE Eötvös Loránd University Budapest Hungary; ^2^ Department of Anatomy, Cell and Developmental Biology ELTE Eötvös Loránd University Budapest Hungary

**Keywords:** condition‐dependence, eumelanin, melanocortin hypothesis, migration, molt, pheomelanin

## Abstract

Sexually dimorphic ornamental traits are widely regarded as indicators of nutritional condition. However, variation of nutritional condition outside the reproductive and the ornament production seasons has rarely been considered, although it affects the generality of information content, especially for ornaments that may be used across the year. We measured several indicators of migratory and molt condition in male and female blackcaps (*Sylvia atricapilla*) during their autumn migration, and quantified their crown reflectance. We detected robust correlations between migratory and molt condition indices, and the correlation structure was similar in the two sexes. Furthermore, the across‐season measure of body condition was positively related to the darkness of the black crown in males, while being unrelated to reflectance traits of the reddish crown in females. Our results reinforce the possibility that some melanin‐based ornaments may be year‐round indicators of individual quality via their dependence on nutritional condition.

## Graphical abstract

We found a positive correlation between the expression of a melanin‐based plumage ornament and an across‐season measure of body condition. These findings support a mixed interpretation of the information content of melanin ornamentation, with the simultaneous presence of condition‐dependence and pleiotropy.

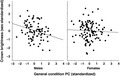

## 1 INTRODUCTION

Condition‐dependence is a fundamental concept in sexual selection research. Costs of mate choice (in terms of time, energy, and opportunity) necessitate benefits that outweigh these costs. A major group of benefits arises from condition‐dependence, which means the adjustment of signals to the physiological state of the individual, as a consequence of the costs of expressing elaborate signal values (Penn & Számadó, 2020). Condition‐dependence may link signal expression to the additive genetic background of condition (Rowe & Houle, 1996), thereby building a connection between mate choice and adaptation to the prevailing environment (Lorch et al., 2003), at the same time also lessening the chance that directional selection by mate choice depletes the additive genetic variance of the signal (Tomkins et al., 2004).

Recent research of condition‐dependent signals has brought new suggestions for general mechanisms connecting organismal performance and signal expression (Emlen et al., 2012; Koch et al., 2017; Lailvaux & Irschick, 2006). Another promising direction might be the use of overarching, multidimensional approaches to condition (Milot et al., 2014). At the same time, a tendency is seen for the preservation (Labocha & Hayes, 2012), reinstatement (Schulte‐Hostedde et al., 2005), and reinterpretation (Salin et al., 2015) of classical condition measures. All of these developments make sense only if we (a) clarify the timing and mechanism of signal production and (b) also take into account the possibility that signal expression may feed back to condition, for example, through resource acquisition (Klug et al., 2010) or aggression by conspecifics (Webster et al., 2018).

In studies of the condition‐indicator value of plumage signals in migratory birds, several recent developments can be observed, and these developments seem to promote an increased coverage and also an increased integration across time and space. For example, the dependence of signal expression on the environment encountered in the nonbreeding season has repeatedly been confirmed (Hargitai et al., 2012; Saino et al., 2015). Moreover, the dynamic dimension of the condition‐signal link has received increased attention for two reasons. First, signal deterioration has been highlighted as a possible condition indicator (Hegyi, et al., 2019; Surmacki et al., 2011). Second, signals have been demonstrated to indicate the trajectory of condition change (Hegyi, et al., 2019).

Plumage signals are produced at one or two yearly molts, and this reinforces the question of how reliably these signals can indicate individual quality (Wilson & Nussey, 2010) rather than only temporary condition at the time of signal production. There are some straightforward links to general quality (e.g., food acquisition for carotenoid‐based traits, Olson & Owens, 1998), but such mechanisms are unknown or debated for some other signal types. In particular, condition‐dependence is an extremely controversial topic for melanin‐based ornaments (San‐José & Roulin, 2018; see Section 4 for more details). Furthermore, the complete postbreeding molt is a particularly likely time for the production of plumage signals, but these are thought to be used principally during the next breeding season. Early production leaves ample time not only for signal deterioration (Delhey et al., 2010) but also for signal functioning outside the breeding season (Lemel & Wallin, 1993; Queller & Murphy, 2017). This in turn leads us back to our previous question on the general quality indicator value of plumage signals. Do these indicate actual condition outside the breeding or molting season? This topic has rarely been examined in the field (Chui et al., 2011; Dias et al., 2016; Peiró & Pagani‐Núñez, 2016). Even less is known about the interdependence of condition metrics across the yearly cycle.

**FIGURE 1:**
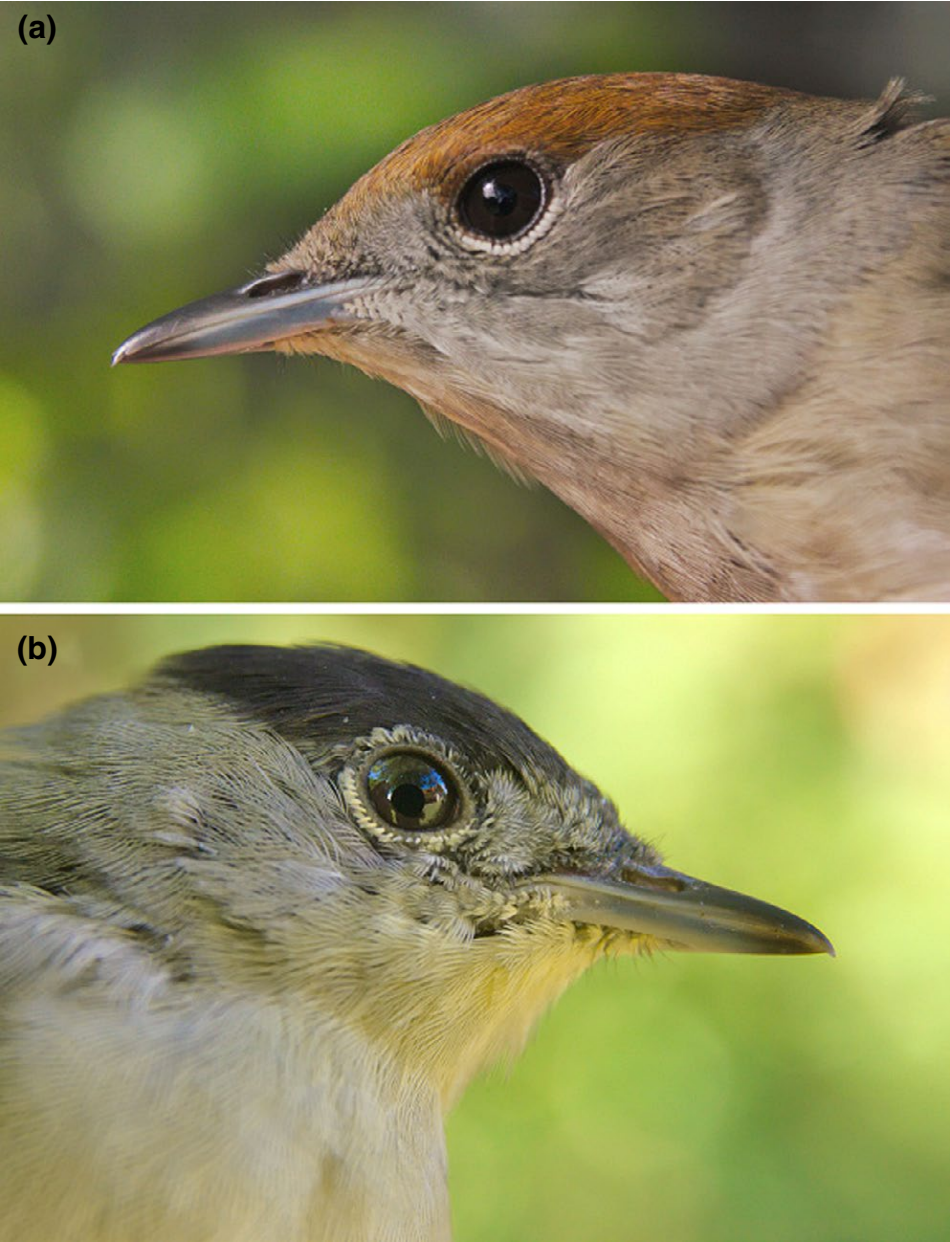
The appearance of the ornamental crown in (a) female and (b) male blackcaps. Photographs by Miklós Laczi

Here, we examine aspects of cap reflectance in male and female blackcaps (*Sylvia atricapilla*) during their autumn migration (i.e., after the complete summer molt) in relation to aspects of body condition during the summer molt and actual body condition at migration. Blackcap cap reflectance has rarely been examined as a signal trait and little is therefore known about its phenotypic variability and possible information content (but see Catoni et al., 2009). A particularly interesting aspect of this putative ornamental trait is that it is likely eumelanin‐dominated (black) in males but pheomelanin‐dominated (reddish) in females (Figure 1), and the two pigment types have been suggested to confer contrasting information (D'Alba & Shawkey, 2019; see also Catoni et al., 2009). We first investigate the temporal integration of body condition by looking at the correlation matrix of condition measures and its similarity between sexes. We then estimate the main axes of body condition variation and relate these axes to crown color expression in males and females.

## 2 METHODS

Field data collection was licensed by the Országos Környezetvédelmi, Természetvédelmi és Vízügyi Főfelügyelőség, Hungary (permit number 14/3858‐9/2012).

### 2.1 Field methods

We sampled migrating blackcaps at Ócsa Bird Ringing Station, Hungary (N47.2970, E19.2104; see detailed description in Csörgő et al., 2016) in the autumns of 2014 and 2015. Due to the year‐dependent migration phenology of the species and logistic constraints, field sampling was done approximately every week from mid‐August to late November in 2014 (65 males and 87 females after excluding juvenile birds that had only partly replaced their crown feathers in summer), whereas it covered three capture occasions in late September and early October in 2015 (18 males and 27 females after a similar exclusion). Lack of specific data reduced sample size available for specific analyses. We took standard morphometric data and condition indices routinely taken at ringing stations (wing length using a ruler to the nearest mm, body mass with a spring balance to the nearest 0.1 g, and fat and muscle scores taken visually, Demongin, 2016). We collected the two second outermost rectrices for measurements of feather growth and took spectrometric data from the crown. We measured reflectance using a USB2000 spectrometer, DH‐2000 light source, R400‐7 sensor (oriented at a 90° angle to the surface) relative to a WS‐1‐SS white standard (Ocean Optics Europe). We excluded incoming ambient light from the measured area. We recorded three consecutive spectra from each individual with OOIBase32 software (Ocean Optics Europe) and averaged the three measurements (measurement repeatabilities of spectral variables were high, see Statistical analyses below). For more details of spectral measurements, see Laczi et al. (2017).

### 2.2 Laboratory measurements

One measure taken from the collected tail feathers was the width of 3–5 consecutive growth bars (light and dark) in the middle section of the feather under direct illumination, using a calliper (nearest 0.1 mm). Feather growth bar width is an index of feather growth rate and thereby body condition during molt (ptilochronology, Grubb, 1989). We divided the measured width by the number of the measured bars and averaged the result between the left and right rectrices (correlation between the two sides *r* = .663, *p* < .001, *n* = 229). The second measure taken from these feathers was feather mass, a measure of overall material investment into a single rectrix by the bird (measured with a Mettler Toledo AE200 analytical balance in the laboratory to the nearest 0.0001 g). Feather mass as a second feather growth axis may provide important additional information over feather growth rate, for example, due to the repeatedly demonstrated trade‐off between growth rate and feather quality (e.g., Dawson et al., 2000; Vágási et al., 2012; Szép et al., 2019), which indicates that feather growth alone may provide an incomplete picture of condition at molt.

### 2.3 Statistical analyses

We included body mass, fat score, and muscle score as measures of actual, migratory condition (these convey partly different information; Labocha & Hayes, 2012). As measures of molt condition, we included feather growth rate and feather mass (as discussed above), and also included wing length as a third feather growth measure available as part of the standard field protocol at the ringing station. Age of the bird (first‐year or older as determined in the field) was not considered here as it was not significantly related to any trait we measured here (all *p* > .06). We first constructed the correlation matrix of all six condition measures across seasons for males and females, and compared these matrices between sexes using the software CPC (Phillips & Arnold, 1999). We then ran principal components analyses (PCA) for condition measures according to the results of this matrix comparison. According to a PCA of 20nm spectral bands (Bennett et al., 1997; see Table S1 in the Appendix for details) with the Kaiser criterion (eigenvalue >1), reflectance variation had one dominant descriptor in males (mean reflectance (320–700 nm) or brightness), and two descriptors in females (brightness and brown chroma, *R*_500nm‐700nm_/*R*_320nm‐700nm_). These variables were calculated directly from raw spectral data instead of relying on the results of the spectral band PCA because, while well representing the main directions of reflectance differences, spectral band PCs seem to provide an inaccurate representation of the relative contributions of particular wavelengths to spectral shape variation (Evans et al., 2010). Spectral variables were log transformed before analysis to improve normality. Repeatability of log‐transformed spectral variables was high between consecutive spectra of the same individual (intraclass correlation calculated in rptR in R; male brightness RI = 0.678, *p* < .001; female brightness RI = 0.926, *p* < .001; female brown chroma RI = 0.976, *p* < .001). Before using the first condition PC as an independent variable across sexes, it was standardized (mean of zero, standard deviation of one) for year in females and for the whole group in males. The second condition PC was standardized for year. We finally assessed the relationship between condition PCs and the reflectance measures using Pearson correlations (female brown chroma) and a general linear model (male and female brightness). The latter model consisted of brightness as a dependent variable, sex as a factor, the two standardized condition PCs as continuous predictors, and the two‐way interactions between the sex and the PCs. Statistical analyses were done in Statistica 5.5 (StatSoft, Inc).

## 3 RESULTS

**FIGURE 2:**
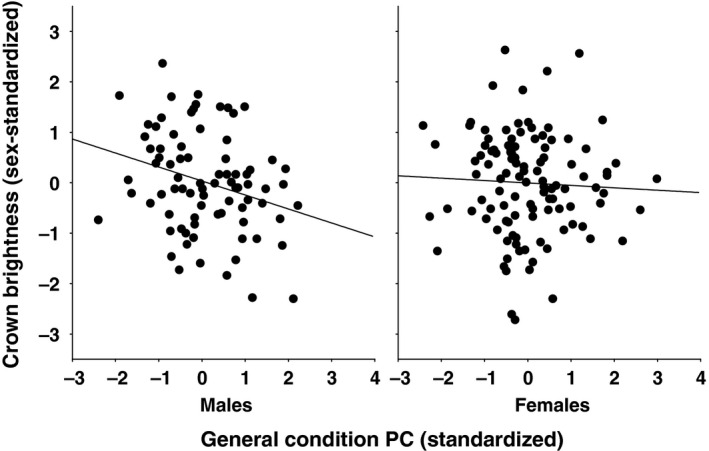
The sex‐dependent relationship between the across‐season condition PC and crown brightness in blackcaps during autumn migration

Matrix proportionality was the best model between the correlation matrices of condition measures of the two sexes, indicating that all PCs were shared between the sexes (Table 1). Therefore, we ran a pooled PCA for the two sexes. Using the Kaiser criterion, this analysis yielded a first PC that was positively correlated with all condition measures, and a second PC that was positively related to measures of molt condition (feather growth rate, feather mass, wing length) but negatively to measures of migratory condition (fat score, muscle score, and body mass) (Table 2). We used these PCs as the independent variables in the following analyses. Neither PC (both year‐standardized) was significantly related to brown chroma in females (PC1 *r* = .090, *p* = .351, *n* = 109; PC2 *r* = −.022, *p* = .814, *n* = 109). PC1 (standardized as described in the Methods) showed a significant interaction with sex in determining crown brightness (Table 3, Figure 2). The relationship was significantly negative in males (*F*_1,79_ = 6.66, *p* = .012), but not significant in females (*F*_1,107_ = 0.24, *p* = .625). PC2 (year‐standardized), on the other hand, showed little relationship with crown brightness in either sex (Table 3).

**TABLE 1:** AIC values from common principal components (CPC) analysis testing the level of similarity of the correlation matrices of our condition measures between males and females. This method evaluates a hierarchy of models that represent different degrees of matrix similarity, looking for the number of dominant PC axes (first, first two, first three, etc.) that are shared between two data sets. Very small degrees of similarity are termed an “unrelated structure.” The highest smilarity levels are when all PCs are similar in direction (full CPC), direction and relative importance (matrix proportionality) or direction and absolute importance (matrix equality). To choose the model best supported by our data, we looked for the simplest model within a difference of AIC = 2 from the model with the lowest AIC. Note that this is not necessarily the model with the lowest AIC

Model	AIC
Equality	16.990
Proportionality	18.921
All PCs common	25.888
4 common PCs	27.618
3 common PCs	28.331
2 common PCs	34.275
1 common PC	36.274
Unrelated	42.000

**TABLE 2:** Results of PCA of our blackcap condition measures. The loadings of component variables, eigenvalue, and percent variance explained are shown for the PCs exceeding an eigenvalue of 1

Measure	PC1	PC2
Feather growth rate	0.535	0.311
Feather mass	0.649	0.522
Wing length	0.730	0.346
Fat score	0.664	−0.555
Muscle score	0.255	−0.600
Body mass	0.751	−0.315
Eigenvalue of PC	2.31	1.26
Percent variance explained by PC	38.50	20.94

**TABLE 3:** Crown brightness of male and female blackcaps in relation to the PCs of condition: general linear models with backward stepwise simplification. Statistics for the removed terms reflect their reintroduction to the final model one by one (but together with their component variables in the case of interactions)

Parameter	*df*	*F*
Sex	1, 186	1585.48***
Overall condition PC	1, 186	6.72*
Condition contrast PC	1, 185	0.14
Sex x overall condition PC	1, 186	4.25*
Sex x condition contrast PC	1, 184	0.54

* *p* < .05.

*** *p* < .001.

## 4 DISCUSSION

We emphasize three important points from our results. First, the analyses suggested a single overarching axis of body condition across seasons in blackcaps. Second, this overall condition axis positively correlated with crown darkness in males. Third, this relationship was absent in females.

### 4.1 Body condition differences and their stability

The robust correlations between summer molt condition and autumn migratory condition (for previous suggestions of such a pattern, see Minias et al., 2014; Pérez‐Arteaga et al., 2019; Ruhs et al., 2019) should be interpreted in light of what we know about nutritional reserve management and year‐round environmental preferences in this migratory songbird. This species has a mixed reserve management strategy during migration, meaning that it does not accumulate the greatest amount of fat until just before crossing the Sahara (Ozarowska, 2015). Therefore, the reserve state we see in Central Europe during the autumn migration is not at its maximum. This is important as it may allow greater plasticity in tracking ambient environmental conditions by the physiological state of the individual. This plasticity, however, could either inflate or deflate the correlation between migratory and molt condition, depending on the variation of physiological state and the variability of ambient environments the individual encounters.

From the perspective of environmental variability, the individual may be exposed to a bewildering variety of conditions, leading to gross fluctuations of nutritional state during both migration (Araujo et al., 2019; Guglielmo, 2018) and wintering (Carrascal et al., 1998; Tinkler et al., 2009). From this perspective, the correlation of migratory and molting condition is truly surprising and indicates that a given individual may either not meet such a variety of environments or that some mechanism dampens the effect of such variation on body condition (see e.g., Klinner et al., 2020). Interestingly, very recent evidence suggests that wintering blackcaps adjust their movements to track climatic conditions, even at the cost of gross habitat changes (Fandos & Tellería, 2020). This environmental tracking ability may contribute to stable individual differences in nutritional state across seasons. The across‐season condition correlation suggests that some consistent differences may indeed exist among individuals in resource acquisition that we may tentatively interpret as manifestations of individual quality (Morehouse, 2014; Wilson & Nussey, 2010). This lends special weight to the correlation between ornamentation and this across‐season condition axis.

### 4.2 Condition‐dependence versus pleiotropy in melanin‐based color

The across‐season condition index was negatively related to black cap brightness in males. There are individual descriptors of both migratory and molting condition that show significant or marginal correlations with male cap brightness (see Table S2 in the Appendix for details). This implies that males with a stronger expression of melanin‐based coloration (darker black) show better nutritional condition across seasons, that is, color expression is positively related to body condition. This agrees with the dominant, positive direction of relationships between melanin‐based color expression and body mass as previously collated from the literature (Guindre‐Parker & Love, 2014; San‐José & Roulin, 2018). Although the literature seems to be loaded with publication bias (Guindre‐Parker & Love, 2014), so the strength of the relationship should be interpreted with caution, its overall direction is likely correct and is very interesting.

The melanocortin hypothesis (Ducrest et al., 2008; San‐José & Roulin, 2018) has been proposed as the default framework to interpret relationships between melanin color and other phenotypic traits. This hypothesis states that melanocortin peptides play roles in the regulation of melanin synthesis, but also many other physiological pathways, giving rise to a multitude of associations between melanin‐based color expression and aspects of physiology, life history, and behavior due to this common regulatory background. Melanocortin‐based regulation predicts a positive association of melanin‐based color expression with metabolic rate, and a negative association with food intake and body mass (Ducrest et al., 2008). For body mass, this is the opposite of what we and most other studies have found (Guindre‐Parker & Love, 2014; San‐José & Roulin, 2018). What makes this even more interesting is that other predictions of the melanocortin hypothesis, including relationships with metabolic rate and food intake (which are possible determinants of condition), are supported, sometimes overwhelmingly so, by literature evidence (San‐José & Roulin, 2018).

These patterns suggest that a mixed interpretation of melanin‐based coloration may be necessary. The melanocortin pathway seems essential for interpreting the links of this ornament type with life history traits, hormones, metabolism, and behavior. Concerning condition‐dependence, we suggest that the production and wearing costs of melanin‐based ornamentation ensure that melanin‐based ornaments cannot be highly expressed by individuals of poor nutritional state, and they may therefore be reliable signals of condition. Multiple potential pathways facilitating the honesty of melanin‐based coloration as an indicator of nutritional state have been suggested (for example, increased attack rate by conspecifics, limitation by trace metals, limitation by essential amino acids that are used for, for example, antioxidant synthesis, and regulation by testosterone; reviewed by, for example, Galván et al., 2015; Griffith et al., 2006; Jawor & Breitwisch, 2003; McGraw, 2008; Poston et al., 2005). However, one question that we still need to answer concerns the strong additive genetic determination of melanin‐based color (Roulin & Ducrest, 2013). Does this preclude condition‐dependence? We suggest that strong heritability can be perfectly compatible with condition‐dependence, if the “good genes” or “genic capture” mechanism operates in melanin‐based traits, that is, if some of the additive genetic background of ornamentation is provided by the additive genetic background of body condition (Penn & Számadó, 2020; Rowe & Houle, 1996; Tomkins et al., 2004). Indeed, this mechanism has been suggested as a potentially general “ingredient” of sexual trait honesty because it simultaneously also resolves the “lek paradox,” that is, the depletion of additive genetic variation due to directional sexual selection, as the additive genetic basis of condition is multigenic and cannot easily be depleted (Tomkins et al., 2004). Therefore, it would be surprising if additive genetic differences in body condition were not indicated by melanin‐based coloration, which is a common ornamentation type across animals (D’Alba & Shawkey, 2019).

### 4.3 Sex difference in condition‐dependence: function or proximate determination?

Our third major result is that female crown color (both brightness and brown chroma) was unrelated to our main condition axis. Interestingly, our finding is consistent with the stimulatory effects of experimental antioxidant intake on male but not female crown color in the same species (Catoni et al., 2009). The apparently different information conveyed by male and female crowns could be explained by as at least two major differences. The first is the potentially different selection pressures on coloration in females than in males, which may include both natural selection, that is, different activity around the nest and the associated different predation pressure, and sexual selection, that is, a different role of crown coloration in males and females (Tobias et al., 2012). More studies of parental care, and especially of visual signaling, are necessary to evaluate sexual and natural selection on crown color in this species. It is, however, certain even at this point that predation and associated adaptations of parental activity are dominant determinants of fitness in blackcaps (Leniowski & Wegrzyn, 2018; Remeš, 2005), providing a logical starting point for such studies.

The second difference is the pigmentary basis of crown color. Based on its appearance, it is likely dominated by eumelanin in males (black) and pheomelanin in females (rufous; D’Alba & Shawkey, 2019). The two types of pigments have been suggested to convey different information due to the different constraints associated with their production, but the role of these differences is currently controversial and difficult to judge. For example, pheomelanin production requires cysteine, an essential amino‐acid necessary for antioxidant synthesis to fight oxidative stress (Galván et al., 2015), but it also sequesters excess cysteine generated by physiological stress (Galván et al., 2012). The greatest difficulty concerning pheomelanin and eumelanin is that they nearly always occur together in the integument of birds and mammals (D’Alba & Shawkey, 2019), and it has repeatedly been suggested that only one of the two melanin types is responsible for the information content of the given ornament of mixed pigment content (e.g., Arai et al., 2019; Leclaire et al., 2019; Saino et al., 2013). Therefore, a major task with respect to sexual dichromatism in the blackcap crown and its information content will be to clarify the relative roles of pheomelanin and eumelanin in generating phenotypic variation in color (and not only dichromatism).

### 4.4 Future directions

Much research of melanin ornaments and condition is needed, and we suggest three especially promising avenues in general terms. The first is to seek further evidence that the apparent “local” violation of the melanocortin framework in the case of nutritional condition is indeed “local,” that is, other predictions of the framework are indeed robustly supported while the one concerning nutritional state is indeed robustly not, which is essential to clarify the potential information content of melanin ornaments (San‐José & Roulin, 2018). The second is to conduct targeted quantitative genetic studies (e.g., pedigree analyses and breeding or cross‐fostering experiments) to examine additive genetic links between melanin‐based traits and some aspect of nutritional condition (or a related variable, see Hill, 2011). To the best of our knowledge, studies of genic capture (e.g., Birkhead et al., 2006; Delcourt & Rundle, 2011; Herdegen & Radwan, 2015; Kotiaho et al., 2001; Parker & Ligon, 2007) are so far missing for melanin‐based ornaments. The third is to clarify, in as many species as possible, the ratios of eu‐ and pheomelanin in coloration and their relative roles in mediating ornament information content (D’Alba & Shawkey, 2019). This may be especially fruitful in species with sexual dichromatism in these ratios, like the blackcap. Returning to our study system, studies of sexual selection in blackcaps have been, to the best of our knowledge, limited to the roles and information content of song (e.g., Hoi‐Leitner et al., 1993, 1995; Leedale et al., 2015; Linoissier et al., 2015). Our study suggests that a new, promising avenue would be to examine the phenotypic variation of crown color as a sexually dichromatic plumage trait, and the background, information content and function of this variation.

## CONFLICTS OF INTEREST

The authors declare no conflicts of interest.

## AUTHOR CONTRIBUTION

**Gergely Hegyi:** Conceptualization (equal); Data curation (equal); Formal analysis (equal); Funding acquisition (equal); Investigation (equal); Methodology (equal); Project administration (equal); Supervision (equal); Writing‐original draft (equal); Writing‐review & editing (equal). **Miklós Laczi:** Conceptualization (equal); Data curation (equal); Formal analysis (equal); Investigation (equal); Writing‐review & editing (equal). **Dóra Kötél:** Data curation (equal); Investigation (equal); Writing‐review & editing (equal). **Tibor Csörgő:** Data curation (equal); Investigation (equal); Writing‐review & editing (equal).

## Supporting information

Appendix S1

## Data Availability

Melanin‐based ornament darkness positively correlates with across‐season nutritional condition: Dryad https://doi.org/10.5061/dryad.z612jm69d.
