# Supplementary material for: Melanin‐based ornament darkness positively correlates with across‐season nutritional condition
Source: Ecol Evol. 2020 Oct 12;10(23):13087–94. doi: 10.1002/ece3.6898 (PMC7713921; doi:10.1002/ece3.6898)
Supplement: Supplementary file 1 — Appendix S1 [file ECE3-10-13087-s001.docx]

Appendix, Table S1. Results of PCAs of blackcap crown reflectance using 20nm wavelength bands, conducted separately for the two sexes. PCs exceeding an eigenvalue of 1 are shown

| Measure | Male PC1 | Female PC1 | Female PC2 |
| --- | --- | --- | --- |
| Loading 320-340nm | 0.939 | 0.808 | -0.502 |
| Loading 340-360nm | 0.969 | 0.873 | -0.442 |
| Loading 360-380nm | 0.974 | 0.900 | -0.415 |
| Loading 380-400nm | 0.982 | 0.925 | -0.372 |
| Loading 400-420nm | 0.985 | 0.937 | -0.341 |
| Loading 420-440nm | 0.987 | 0.949 | -0.290 |
| Loading 440-460nm | 0.991 | 0.957 | -0.244 |
| Loading 460-480nm | 0.994 | 0.967 | -0.185 |
| Loading 480-500nm | 0.995 | 0.980 | -0.112 |
| Loading 500-520nm | 0.998 | 0.992 | -0.011 |
| Loading 520-540nm | 0.997 | 0.989 | 0.085 |
| Loading 540-560nm | 0.995 | 0.979 | 0.171 |
| Loading 560-580nm | 0.993 | 0.964 | 0.211 |
| Loading 580-600nm | 0.989 | 0.947 | 0.255 |
| Loading 600-620nm | 0.987 | 0.935 | 0.326 |
| Loading 620-640nm | 0.981 | 0.907 | 0.413 |
| Loading 640-660nm | 0.965 | 0.868 | 0.469 |
| Loading 660-680nm | 0.952 | 0.846 | 0.498 |
| Loading 680-700nm | 0.951 | 0.841 | 0.514 |
| Eigenvalue | 18.26 | 16.29 | 2.22 |
| Percent variance explained | 96.13 | 85.73 | 11.68 |

Appendix, Table S2. Correlations of male crown brightness with component variables of condition PC1 (n=81)

| Measure | Pearson r |
| --- | --- |
| Feather growth rate | -0.091 |
| Feather mass | -0.327** |
| Wing length | -0.150 |
| Fat score | -0.233* |
| Muscle score | -0.014 |
| Body mass | -0.208† |

†, p<0.07, *, p<0.05; **, p<0.01
